# Screen for modulators of atonal homolog 1 gene expression using notch pathway-relevant gene transcription based cellular assays

**DOI:** 10.1371/journal.pone.0207140

**Published:** 2018-12-12

**Authors:** Xin Zeng, Robert Kirkpatrick, Glenn Hofmann, Didier Grillot, Valerie Linhart, Fabrice Viviani, Joseph Marino, Joseph Boyer, Taylor L. Graham, Quinn Lu, Zining Wu, Andrew Benowitz, Rick Cousins

**Affiliations:** 1 R&D Target Sciences, GlaxoSmithKline, Upper Providence, Collegeville, United States of America; 2 R&D Alternative Discovery and Development, GlaxoSmithKline, Upper Providence, Collegeville, United States of America; 3 R&D Platform Technology Sciences, Drug Design and Selection, GlaxoSmithKline, Upper Providence, Collegeville, United States of America; 4 R&D Flexible Discovery Unit, Villebon-sur-Yvette, Paris, France; 5 R&D Statistical sciences, GlaxoSmithKline, Upper Providence, Collegeville, United States of America; 6 R&D Alternative Discovery and Development, GlaxoSmithKline, Stevenage, Hertfordshire, United Kingdom; University of Minnesota Medical Center, UNITED STATES

## Abstract

Atonal homolog 1 (Atoh1) is a basic helix-loop-helix 9 (bHLH) transcription factor acting downstream of Notch and is required for the differentiation of sensory hair cells in the inner ear and the specification of secretory cells during the intestinal crypt cell regeneration. Motivated by the observations that the upregulation of *Atoh1* gene expression, through genetic manipulation or pharmacological inhibition of Notch signaling (e.g. γ-secretase inhibitors, GSIs), induces ectopic hair cell growth in the cochlea of the inner ear and partially restores hearing after injuries in experimental models, we decided to identify small molecule modulators of the Notch-Atoh1 pathway, which could potentially regenerate hair cells. However, the lack of cellular models of the inner ear has precluded the screening and characterization of such modulators. Here we report using a colon cancer cell line LS-174T, which displays Notch inhibition-dependent *Atoh1* expression as a surrogate cellular model to screen for inducers of Atoh1 expression. We designed an *Atoh1* promoter-driven luciferase assay to screen a target-annotated library of ~6000 compounds. We further developed a medium throughput, real-time quantitative RT-PCR assay measuring the endogenous *Atoh1* gene expression to confirm the hits and eliminate false positives from the reporter-based screen. This strategy allowed us to successfully recover GSIs of known chemotypes. This LS-174T cell-based assay directly measures *Atoh1* gene expression induced through Notch-Hes1 inhibition, and therefore offers an opportunity to identify novel cellular modulators along the Notch-Atoh1 pathway.

## Introduction

Notch signaling controls cell fate decisions during development and tissue regeneration. [1, 2] Disruption of Notch signaling, as a result of genetic mutations in Notch or Notch pathway components, is associated with a wide spectrum of human diseases, including hearing loss. [3] The effect of Notch activity on hearing is mediated through the bHLH transcription factor Atoh1. In the mammalian inner ear, the cochlea of homozygous *Atoh1* mutant mice lack differentiated hair cells and associated molecular markers. [4, 5] *Atoh1* S193A mutant mice exhibit cochlear hair cell degeneration and develop profound hearing loss. [6] Conversely, forced overexpression of Atoh1 in the vestibular or cochlea in perinatal or mature animals induces reprogramming of the supporting cells in the cochlea resulting in the generation of supernumerary hair cells. [7–9] These observations suggest that increased Atoh1 expression could potentially be beneficial to restore hearing upon hearing loss, a prevalent healthcare concern during aging and after acoustic trauma.

Atoh1 expression is normally tightly regulated by Notch signaling during development. The activation of Notch by its ligands expressed from adjacent cells induces the sequential proteolytic cleavage of the Notch receptor, first by ADAM17 and then by γ-secretase. [10] This results in the release and subsequent translocation of NICD to the nucleus where it activates Hes1 gene transcription, which prevents *Atoh1* gene transcription by binding specifically to the Hes1 site in the *Atoh1* promotor element. [10] Therefore, Notch inhibition relieves the repression of Hes1 and induces Atoh1 expression. Indeed, it has been shown that inhibition of Notch signaling causes upregulation of Atoh1 expression and increases the number of embryonic cochlear hair cells at the expense of supporting cells, even in postnatal animals following acoustic trauma-induced hair cell loss. [11–14]

Over the past 20 years, the identification and the development of small molecule Notch inhibitors has largely been focused on the γ—secretase inhibitors (GSIs) which inhibit the proteolytic cleavage of Amyloid Precursor Protein (APP) by γ-secretase complex. [13] This cleavage leads to the generation and release of the Aβ peptide, in particular the 40 and 42 isoforms (Aβ40 and Aβ42), aggregation-prone products that accumulate in the brains of Alzheimer’s Disease (AD) patients. [13] While numerous γ-secretase inhibitors (GSI) have been developed to treat AD, the therapeutic potential of these inhibitors has been limited due to their toxicity in gut and the immune system, most likely owing to the on-target side effects of Notch inhibition. [3] Consequently, recent efforts on GSIs have been focused on developing the so called “Notch-sparing” compounds (γ-secretase modulators, GSM) that selectively inhibit γ-secretase activity toward APP processing, but with attenuated activity toward Notch inhibition, such as E2012. [13, 15]

Motivated by the observations that Notch inhibitors promote sensory hair cell growth in the inner ear in animal models, we decided to screen for modulators of the Notch-Atoh1 pathway, which is relevant to hair cell regeneration. To do this, a scalable cellular system that links Notch to Atoh1 expression is necessary. This has been complicated since the inner ear cells are sparse and are extremely difficult to isolate and maintain in tissue culture at a scale that is suitable for testing batches of small molecules. As an alternative, we turned to the gut epithelium, where Notch-Atoh1 is involved in the secretory cell lineage decision during normal regeneration from crypt cells. [16] We identified a colon cancer cell line LS-174T, which increases the expression of endogenous Atoh1 upon Notch inhibition. An *Atoh1* promoter-driven NanoLuc reporter assay using the LS-174T cell line was used to screen a target annotated compound library (6000 compounds, ~700 targets) [17–19] to identify *Atoh1* gene transcription inducerss. To confirm hits from the reporter assay screen, a medium-throughput gene transcription assay was developed to detect endogenous *Atoh1* gene expression by RT-qPCR. Notch inhibitory GSIs were readily identified by this screening strategy and these studies demonstrate that the LS-174T cell line is a valid model in which to study signaling events downstream from Notch inhibition leading to Atoh1 induction. Unlike the previously reported Notch assays which measure the Notch NICD-driven responsive reporter activity, [20, 21] the RT-qPCR assay directly measures endogenous Notch signaling in a relevant cellular context and therefore offers a more direct approach to screen for Notch pathway inhibitors.

## Materials and methods

### Cell lines and tissue culture

Human colon cancer cell line LS-174T (CL-188) was obtained from the ATCC. All cell culture supplies were from ThermoFisher. The cells were cultured at 37°C in RPMI-1640 media (Cat#12633–012) supplemented with 10% Fetal Bovine Serum (Cat#10099–141), and 1% Glutamax (Thermofisher, Cat# 35050–61) at 60% confluency at passages between 3 to 25. Human recombinant R-spondin-1 was purchased from Sigma (SRP3292).

### Compounds

Putative benzodiazepine GSI compounds were from an internal GSK collection (Compound I, CAS No. 504428-17-5 and Compound III, CAS No. 209984-56-5) or purchased (Compound II, CAS No. 959977-22-1). Putative gamma-secretase modulator, E-2012 (Compound IV, CAS No. 870843-42-8) and GlaxoSmithKline (GSK) chemical compounds (Compound V-VII) were from an internal GSK compound collection. Protease inhibitors Compound VIII (BMS 566394, CAS No. 503166-51-6) prepared per reported synthesis, [22] whilst Compound IX (GW280264X, CAS No. 866924-39-2) was from internal collection and Compound X (TAPI, CAS No. 163958-73-4) was purchased from commercial supplier (Calbiochem). All compound stocks were prepared in DMSO at 10mM and dispensed into the assay-ready plate using Echo (Labcyte). The assay plates were stored at -20 °*C* and warmed to room temperature for cell plating.

### Atoh1 immunostaining assay

Immunofluorescent staining for Atoh1 was performed using anti-Atoh1 antibody (5ug/ml) (PAS 29392, ThermoFisher) based on the standard protocol. [18] Briefly, the LS-174T cells were diluted in RPMI-1640 media supplemented with 10% FBS and plated at 5000 cells/well in 384 black-walled, clear-bottom, Poly-D-Lysine coated plates (Greiner Bio-One, 781946) that were pre-stamped with compounds. The plates were incubated at 37 °C, 5% CO_2_ for 72hrs. The media was removed using a BioTek ELX450 plate washer (BioTek) and cells were fixed in 3.7% paraformaldehyde (diluted from 37% formaldehyde, VWR Scientific, JT2106-3) for 10min, then incubated in blocking buffer consisting of 1X Dulbecco’s Phosphate Buffered Saline (D-PBS) with 0.2% TritonX-100 (D-PBST) supplemented with 3% BSA blocker solution (10% BSA blocker is from Thermo Scientific 37525) for 2hrs. The cells were then incubated with Atoh1 antibody (PAS-29392, ThermoFisher) (prepared in blocking buffer at 1:500 dilution) overnight at 4 °C. The plates were washed in PBST, 5 x 5min each followed by incubation with secondary Goat Anti-Rabbit IgG (FITC conjugated) (1:5000 dilution) for 1hr, washed in D-PBST and incubated with Hoechst 33342 dye for 10 min before imaging. The fluorescent signal is stable for a few days stored in dark. All aspiration and dispensing was performed with a BioTek plate washer and Multidrop Combi (Thermo Electron Corporation, Waltham, MA), respectively.

The blocking peptides derived from Atoh1 amino acid sequences 285–314 (peptide 1), 305–334 (peptide 2) and 325–354 (peptide 3) EENSKTSPRSHRSDGEFSPHSHYSDSDEAS) covering the immunogenic region used to generate Atoh1 antibody were synthesized (21^st^ Century Bio). The peptide (10mg/ml, 2000x fold excess) was mixed with the antibody before incubating with the fixed cells. The scrambled peptide, SASNQLKPGIMQLPESMQVSNSLPKRPEST was used as a negative control.

The Atoh1 immunofluorescent imaging quantification and analysis were carried out as previously described. [18]

### Cell lysis and Turbocapture plate-based RNA purification

LS-174T cells cultured in 384-well plates were lysed with 30ul of TCL buffer from the Turbocapture 384mRNA kit (Cat# 72271, Qiagen) and 25ul of lysates were transferred to 384-well Turbocapture plate, incubated for 1hr at RT. The plates were washed and eluted in 30ul nuclease-free water at 65 °C for 5min per the manufacturer’s instructions. All liquid handling was carried out using Cybi dispenser (Cybio) for plate transfer, Multi-drop Combi dispenser (Thermo) for wash buffer and elution buffer dispensing, and the BioTek plate washer for aspiration. The mRNA purified on-plate was either stored at -80 °C or used for reverse transcription.

### One-step Real-time RT-qPCR analysis

The one-step Real-time RT-qPCR reaction was set up using the light cycler 480 RNA master hydrolysis probes kit (Cat# 04991885001, Roche Applied Sciences). Primers for *Atoh1* were custom made by IDT as follows: PrimeTime Primer 1: GGG AGA GAA GGA GAC AAA TTC TT, PrimeTime Primer 2: TTC TGC GGG AGG GTA CT, PrimeTime Probe: PRB-/56-FAM/AA AGT CGA G/Zen/A AGTGCA GAG CGT CC/3IABkFQ/ PrimeTime; The primer and probes for the Hes-1 (Hs00172878_m1), Myc (Hs00153408_m1), DKK1 (Hs00183740_m1), RPL13A (03043885_g1) and Axin2 (Hs00610344_m1) were purchased from ThermoFisher.

Probes (250nl/well) and mRNA (500nl/well) were dispensed into the PCR plate by Echo 555 (Labcyte). RT-qPCR Master mix was dispensed by multi-drop Combi nl (Thermo). The final reaction volume was 5ul. The plates were spin-mixed and sealed and run on the Lightcycler 480 (Roche). When working with multiple plates, RNA was converted to cDNA by running all plates through reverse transcription protocol first (3min at 37 °C). Then the plates were stacked on the CRS catalyst (Thermo) for automated feeding onto to the Lightcycler 480 for Real-time PCR reactions.

### RT-qPCR data analysis

The CT (Crossing Point) obtained from the Lightcycler was converted to ΔCT by using the formula ΔCT = CT DMSO (robust mean)-CT Sample. ΔCT value was used as the response for curve fitting for pEC50 or IC50 calculations in R using the drm function of the drc package to fit a four-parameter logistic function (L.4 in the drc package) of the form

$$\Delta \text{CT}=\text{Max}+\left(\text{Min}-\text{Max}\right)/\left(1+10^{\left(\text{Slope}\;\text{X}\;\left(\text{Log}\;10\left(\text{Compound}\;\text{Concentration}\right)+\text{pXC}50\right)\right)}\right)$$

For some plots, ΔCT value is converted to fold of induction (or inhibition) by the formula fold of induction = 2^ΔCT^. The data were graphed using Spotfire (Tibco Software, Inc) or in GraphPad (Prism).

### *Atoh1* reporter assay

*Atoh1* promoter region -1031bp to -1bp was subcloned into the lentiviral vector pLEXmcs-NanoLucPEST plasmid to replace the CMV promoter. This promoter contains 3 *Hes1* binding sites (CACGCG at -305, -269, -159). The *Atoh1* mutant has all three sites mutated to GTCGAC. The lentivirus was packaged using the Thermo Scientific Open Biosystems TransLenti Viral ORF Packaging System (TLP4616, TLP4617). The virus was titrated and aliquoted to store at -80 °C.

For transductions, the lentivirus was thawed and added 10% (v/v) to LS-174T cells suspended in culture media at 1.6e5 cell/ml. The cells were plated 30ul/well (5000 cells/well) in 384-well plates (Greiner white LV highbase TC plate, #784084) pre-stamped with 300nl of compounds. Cells were incubated with 37°C for 48hrs. The Nano-Glo substrate (N1120, Promega) was added to cells according the manufacturer’s instructions. The Luciferase activity was measured on a Viewlux (Perkin Elmer). The single point (primary screen) and the full dose response of the luciferase assay data were analyzed with AbaseXE (IDBS) and visualized by Spotfire (Tibco Software, Inc) or in GraphPad (Prism).

### Compound screen and dose response

The library used in this screen is an internal GSK collection of 5857 biological target-annotated compounds (Biologically Diverse Compound Set, BDCS). The compounds were transferred to 384-well Greiner plate at 300nl/well using Echo acoustic dispenser (Labcyte). Column 6 (all 16 wells) contained DMSO only (low control, 0% inhibition). Column 18 (16 wells) contained compound II at 1mM concentration in DMSO (high control). 30ul of cells in the culture media was dispensed onto the compound plate (1:100 dilution of the compounds, 1% DMSO final concentration). Compounds were tested as dose response starting at a stock concentration of 10mM and diluted serially 3-fold across 11 points, also in DMSO.

## Results

### γ-secretase inhibitors induce *Atoh1* mRNA in LS-174T cells

In order to establish a cellular model to study Atoh1 induction, we obtained a number of inner ear cell lines derived from the embryonic stages of the rodent inner ear and immortalized by the SV40 T antigen (US/VOT-E36, [23] UB/OC-1, [24–26] UB/UE-1 [27, 28]). However, none of these cell lines were found to express Atoh1 in response to Notch inhibition (data not shown). By examining gene expression in a panel of colon cancer cell lines, we found that LS-174T cells express Atoh1 and that this expression can be modulated by Notch signaling, making it suitable for studying Notch signaling-dependent *Atoh1* transcription. [29]

**Fig 1:**
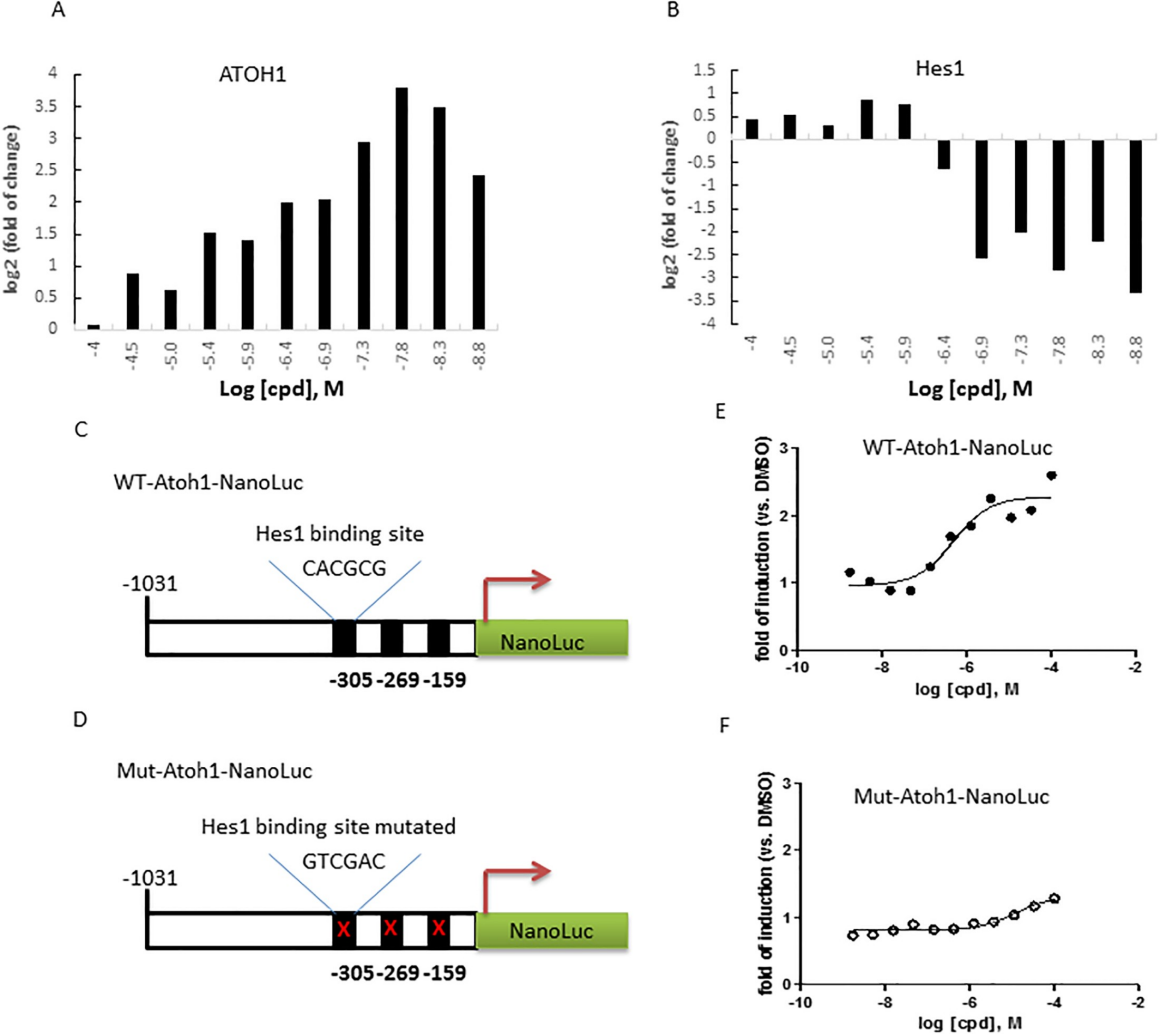
Validating the effect of GSI tool compound on *Atoh1* and *Hes1* gene expression and the *Atoh1* promoter driven reporter in LS-174T cells. A and B. The endogenous *Atoh*1 and *Hes1* gene expression in LS-174T cells upon GSI compound II treatment was detected by Real-time RT-qPCR. LS-174T cells were treated with compounds at doses indicated for 72hrs and mRNA was extracted for RT-qPCR analysis using the corresponding primers against *Atoh1*, *Hes1* and *GAPDH* (housekeeping control, not shown). C and D. The design and validation the *Atoh1* promoter-driven NanoLuc reporter assay. The WT *Atoh1* promoter (-1031 to +2 of the endogenous *Atoh1* gene regulatory region) was subcloned upstream of the NanoLuc luciferase reporter construct (WT-*Hes1*-NanoLuc) (C) in a lentiviral vector backbone. The 3 copies of the *Hes1* binding element CACGCG in the WT *Atoh1* promoter region were mutated to GTCGAC in the mutant reporter (Mut-*Atoh1*-NanoLuc) (D). E and F. The activities of the tool compound in the WT *Atoh1*-NanoLuc (E) or Mut-*Atoh1*-NanoLuc (F) reporter assays. The LS-174T cells were infected with Lentiviruses encoding either WT or Mut *Atoh1*-NanoLuc reporter and plated on compound pre-stamped assay plate. The cells were lysed for NanoLuc activity measurement after 72hr incubation.

To validate the suitability of the LS-174T cell line to screen for Notch-Atoh1 pathway modulators, we tested compound II (a known GSI, [30]) in the *Atoh1* mRNA induction assay. Cells were treated with compounds over a dose range from 0.6nM to 100uM for 24, 48 and 72hrs and then lysed for RNA purification and RT-qPCR analysis of *Atoh1*, *Hes1* and *RPL13A* (housekeeping) gene expression. We found that 72hrs incubation produced the greatest increases in Atoh1 levels by compound II in a dose dependent manner and the induction of Atoh1 correlated well with the inhibition of Hes1 expression (Fig 1A and 1B). We next confirmed the compound II effect is mediated through the promoter on the *Atoh1* gene. The 5’ region of the *Atoh1* gene ~1.0kb (-1031 to +2) upstream of the transcription initiation site contains 3 copies of *Hes1* binding sites (CACGCG; E-boxes [31]) (Fig 1C). It was previously shown that the luciferase reporter driven by this promoter fragment is suppressed upon Hes1 overexpression. Conversely, the Notch inhibitor, which down regulates the Hes1 gene, leads to increased luciferase activity. [29] These responses are abolished when the *Hes1* binding sites are mutated to GTCGAC. [29] We generated a reporter construct in a lentiviral vector by inserting the 1kb promoter element of the *Atoh1* gene upstream of the NanoLuc gene (WT-*Atoh1*-NanoLuc). The mutated promoter (Mut-*Atoh1*-NanoLuc) was used as a negative control (Fig 1C and 1D). To validate these constructs, we pretreated LS-174T cells with compound II and then infected these cells with lentivirus carrying either the WT-*Atoh1*-NanoLuc reporter or the mut-*Atoh1*-NanoLuc reporter. After 72hrs, the cells were lysed and the NanoLuc luciferase activity was measured. As expected, compound II showed strong induction of WT *Atoh1* NanoLuc activity but not the Mut *Atoh1* Nanoluc activity (Fig 1E and 1F), thus demonstrating that the compound II effect in the *Atoh1* reporter assay was mediated through WT *Hes1* binding sites.

In addition to Notch-Hes1 regulation, it has been reported that *Atoh1* gene transcription can also be regulated through the 3’ element on the *Atoh1* promoter by Wnt/β-catenin signaling pathway in neuroprogenitor cells. [32]. According to this report, β-catenin and TCF/LEF directly binds the 3’ enhancer of *Atoh1* to upregulate its expression level. We tested Wnt/β-catenin-dependent Atoh1 expression by treating LS-174T cells with recombinant R-spondin-1, a soluble Wnt ligand that activates Wnt signaling. [33] We did not observe any change in the expression of endogenous Wnt response genes Axin2, DKK1 and Myc (The Wnt homepage, http://web.stanford.edu/group/nusselab/cgi-bin/wnt/) (S1 Fig) or the expression of Atoh1 after these treatments (S1 Fig). Treatment with another soluble ligand, Wnt3a, also failed to induce Atoh1 expression (data not shown). Similarly, GSI compound II did not activate Axin2, DKK1 or Myc, while the reduction of Hes1 and the increase in Atoh1 gene expression were readily detected (S1 Fig). These data suggest that the Notch inhibitor compound II induces Atoh1 expression independent of β-catenin signaling in LS-174T cells.

### A cell-based reporter screen using Atoh1 promoter-driven luciferase assay

**Fig 2:**
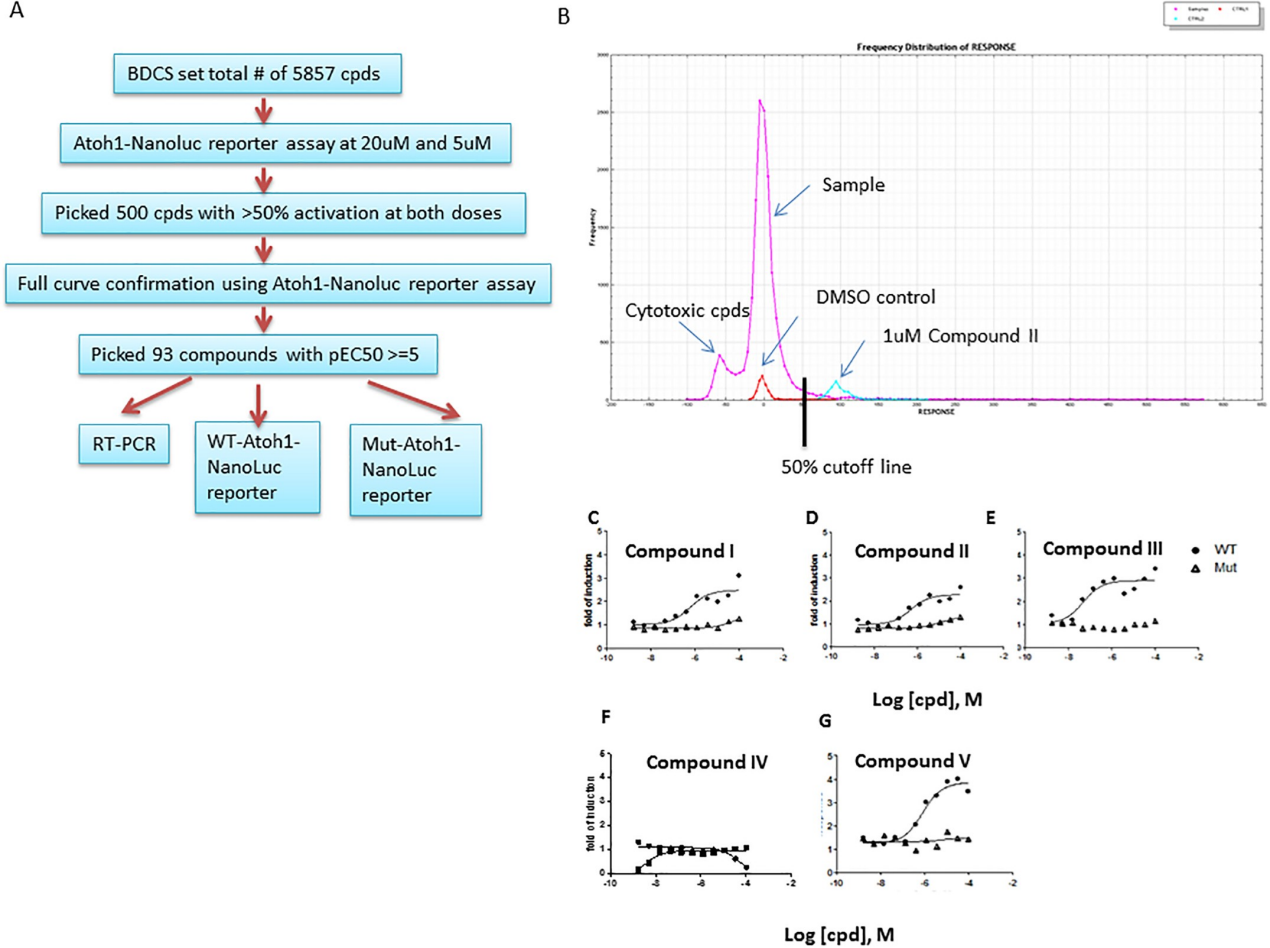
Focused library screen for inducers of *Atoh1* using *Atoh1*-NanoLuc reporter assay. **A.** The screening and hit triage work flow. B. The distribution frequency of the compound activity in the primary screen using 5uM and 20uM as final compound concentrations. The statistical cutoff in the 20uM screen was 55% based on 3X robust standard deviation of the DMSO samples. To be inclusive, the 50% activation was used as cutoff at either dose for selecting compounds for confirmation in full dose analysis in reporter assays (C, D, E, F, G). The data from WT and Mut reporter assays are overlaid and expressed as fold of induction by sample vs. DMSO control.

To identify Atoh1 inducers, we selected the cell-based reporter assay for the primary screen since this format has been widely used in high-throughput screens to identify modulators of gene transcription from a large compound library. Screening-compounds were selected from a collection of annotated libraries comprised of approximately 6000 compounds targeting ~780 known cellular targets. [17] Each target is represented by ~10 compounds. We also added a focused set of 500 compounds that show structure similarity to the known GSIs and 10 putative TACE inhibitors. The screen was performed in 384-well plates and compound concentrations of 5uM and 20uM were used in single point screening (Fig 2A). Data from one whole column (16 wells) of 1uM compound II or DMSO treated cells were used as positive and negative controls, respectively, to calculate the Z’, which ranged between 0.4–0.7. If using the standard statistical cutoff (>3 x standard deviation of the DMSO controls), the cutoff of hits in this screen is 55% activation over DMSO. Given the small library size and to be inclusive, we set the cutoffs at 50% of activation at either dose, resulting in 555 compounds (6% hit rate, Fig 2B). These hits were further tested in full dose response in both WT-*Atoh1*-Luc and mut-*Atoh1*-Luc reporter assays. A total of 93 compounds were confirmed to be selectively active in the WT reporter but not in the mutant reporter (selected curves shown in Fig 2C, 2D, 2E and 2G). The known GSI tools (compounds I-III, XI-XIV) were identified among these hits (Fig 2C, 2D and 2E and S2 Fig). The recovery of the known Notch inhibiting GSIs from the library suggests the screen was robust and effective in identifying Notch inhibitors. In addition, the Notch sparing GSI compound IV was inactive in this assay (Fig 2F), further confirming the validity of the reporter-based screen.

### Independent confirmation of hits in a RT-qPCR and protein immunofluorescent assay for *Atoh1* induction

While the reporter assays are convenient and suitable for high throughput screening of millions of compounds, it is known that luciferase reporters are prone to higher hit rates as false positives may act through target-independent mechanisms. [34] For example, some frequent “hitters” may include compounds that directly bind to luciferase [35, 36] and stabilize its activity. Alternatively, some compounds may nonspecifically activate general transcription or affect cell survival to produce false positive signals.

To confirm the hits and eliminate false positives from the reporter assay, we developed a medium throughput transcription-based assay using RT-qPCR to detect endogenous *Atoh1* induction. RT-qPCR is a well-established method to detect endogenous gene expression which does not require cell engineering. It is highly sensitive, requires small sample input and can be multiplexed, allowing the parallel detection of sets of target genes and controls from a single sample preparation. However, the prohibitive cost of reagents and the tedious RNA purification steps have limited its use in high-throughput applications. Recent improvements in RT-qPCR methodology eliminate the need for RNA purification steps because cell lysis buffers are now compatible with the subsequent enzymatic reactions of RT-qPCR. However, we have obtained variable results using one step cell lysis/RT-qPCR buffers, primarily due to the lack of sensitivity toward targets with low abundance such as *Atoh1*, or to the lack of stability of RNA in the lysates. We therefore utilized the Turbocapture 384-well plates which have OligodT immobilized on the walls of each well, allowing hybridization and attachment of the mRNA on plate from the cell lysates. After washing, the purified mRNA was eluted for subsequent RT-qPCR reactions. The Turbocapture plates are standard 384-well plates and are compatible with standard liquid handlers, including Combi and Bravo. By taking advantage of the aqueous dispensing capability of Echo Acoustic Liquid handling to dispense the cDNAs and primers on a nanoliter scale, we miniaturized the final assay volume to 5ul/well. These modifications allowed the RT-qPCR plates to be handled in batches of 10–30 and stacked on the feeder hotel before starting the heat cycles in a Lightcycler in overnight runs. With this scale, we were able to profile all 93 hits from BDCs and the GSI focused set from the reporter-based screen over a full dose range (11 point) for Hes1, Atoh1 and the housekeeping genes GAPDH or RPL13A as controls. Conventional data analysis for RT-qPCR requires normalization of the target genes to housekeeping genes. However, when PCR is performed in large scale, it tends to generate random errors due to the high sensitivity of the assay. Normalization to housekeeping gene further amplify such errors. So, we decided to curve fit for each individual gene to generate pXC50s without normalization to housekeeping genes. The dose response of housekeeping gene was used as a separate parameter to select hits. The compounds that significantly affected housekeeping gene expression were eliminated.

**Fig 3:**
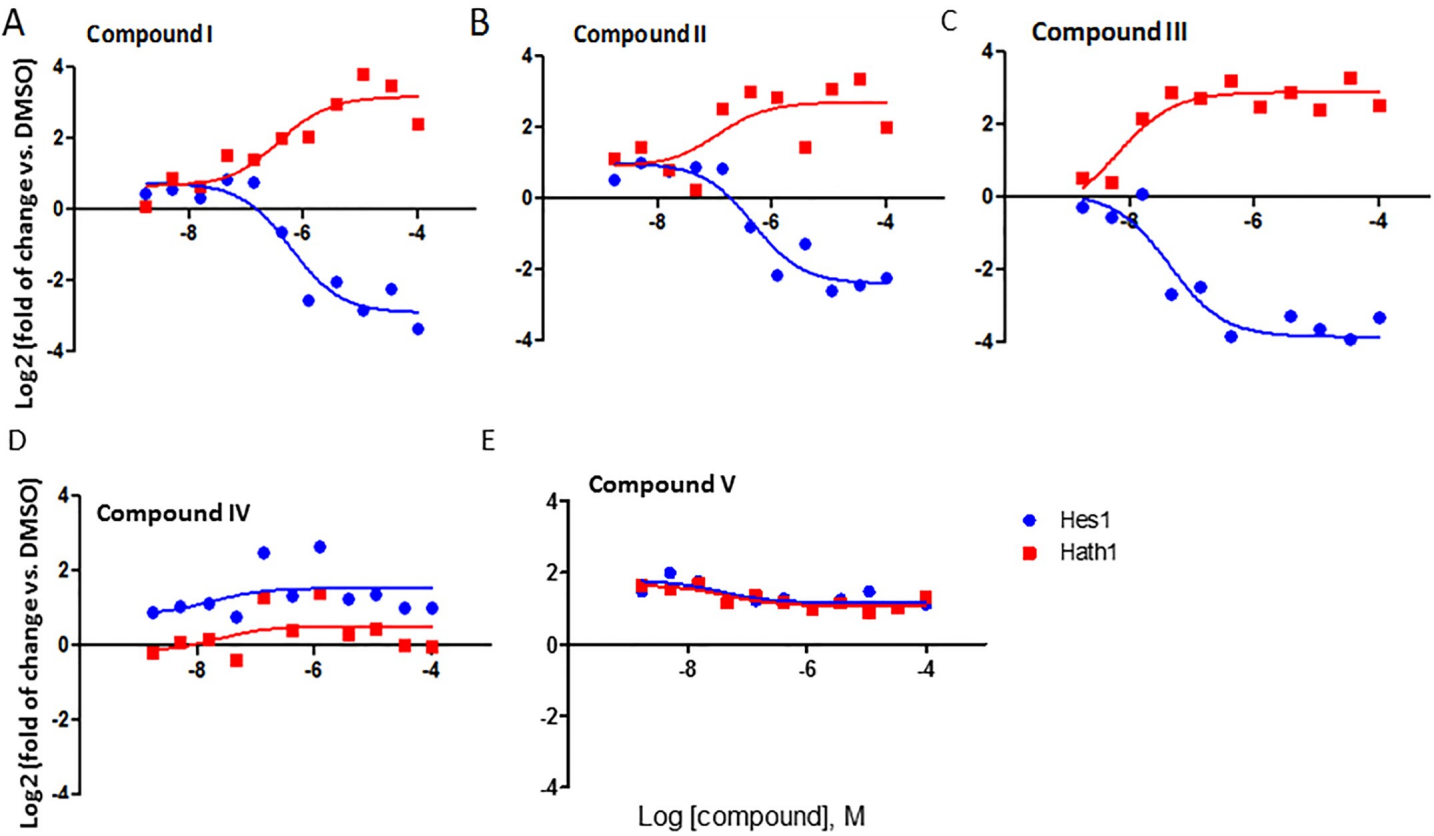
Confirmation of the hits from the Atoh1-Nanoluc reporter screen by RT-PCR for endogenous Atoh1 and Hes1 mRNA. LS-174T cells were treated with compounds for 72hrs and polyA mRNA were extracted on 384-well based Turbocapture plates and used in RT-qPCR analysis for *Atoh1*, *Hes1* and *GAPDH* (for housekeeping, not shown) gene expression. Compound I (A), II (B)and III (C) induced dose-dependent *Atoh1* gene expression, concomitant with the inhibition on *Hes1* gene expression. Compound IV (D) and V (E) were inactive in both *Atoh1* induction and *Hes1* inhibition. The changes in gene expression (>1 for induction) or (<1 for inhibition) are calculated as ΔCT and expressed as log2 (fold of change vs. DMSO).

GSIs included in the screen showed strong *Atoh1* induction and Hes1 inhibition (representative compounds shown in Fig 3A, 3B and 3C and Table 1) as expected. In contrast, TACE inhibitor compounds VIII, IX and X (S4 Fig) and the Notch sparing compound IV (Fig 3E) showed only weak or no activity on *Atoh1* induction and Hes1 inhibition. This may be due to the low expression of ADAM17 in LS-174T cells according the transcriptomics profile of this cell (internal gene expression database). To our surprise, while the GSIs consistently induced *Atoh1* gene expression, the 90 hits from the BDCS that were active in the *Atoh1* reporter assay failed to activate endogenous *Atoh1* gene expression (represented by compound V (non GSI) in Figs 2G and 3E). Consistent with the lack of Atoh1 induction, these compounds also failed to inhibit *Hes1* gene expression (represented by compounds V, VI and VII (non GSIs) in S3 Fig). These data revealed a discrepancy between the reporter assay and the endogenous gene expression assay. The cause of this discrepancy is unclear, but it is not likely due to the direct activation of NanoLuc activity or the activation of general transcription from the basal promoter by the compounds, as these compounds were selective in the WT *Hes1*-NanoLuc reporter activation but not in the Mut-*Hes1* NanoLuc assay. Our data nonetheless reveals the limitations of using cell-based reporter screens and highlights the importance of confirming hits using endogenous gene transcription assays. The medium throughput and cost-effective RT-qPCR protocol developed in this study allows the implementation of this assay early in a prospective screening strategy.

**Table 1:** Summary of γ-secretase inhibitor activities of in *Atoh1* and *Hes1* gene expression (RT-qPCR), Atoh1 protein imaging and *Hes1* promoter driven NanoLuc luciferase reporter assays. Results are listed as pEC50 (*Atoh1* induction in RT-qPCR, imaging and reporter) or pIC50 (*Hes1* inhibition in RT-qPCR) values. The value for RT-PCR, and reporter assay represent the average and the standard deviation of at least 3 independent experiments. The standard error of the imaging assay is based on calculation from the dose response.

compound	*Atoh1 induction (RT-PCR)*		Hes 1 inhibition (RT-PCR)		Housekeeping control (RT-PCR)	Atoh1 induction (protein imaging)		*Atoh1 reporter*		*Atoh1 mutant reporter*
	pEC50	Standard Deviation	pEC50	Standard Deviation	pIC50	pEC50	Standard Error	pEC50	Standard Deviation	pEC50
**Compound I**	5.7	0.17	5	0.12	inactive	7.4	0.74	6.1	1.1	Inactive
**Compound II**	5.6	0.18	5	0.24	inactive	5.9	0.21	6.8	0.5	Inactive
**Compound III**	6	0.14	6	0.31	inactive	5	0.44	7.0	0.5	Inactive
**Compound IV**	<5		<5		inactive	<5		<5		Inactive

**Fig 4:**
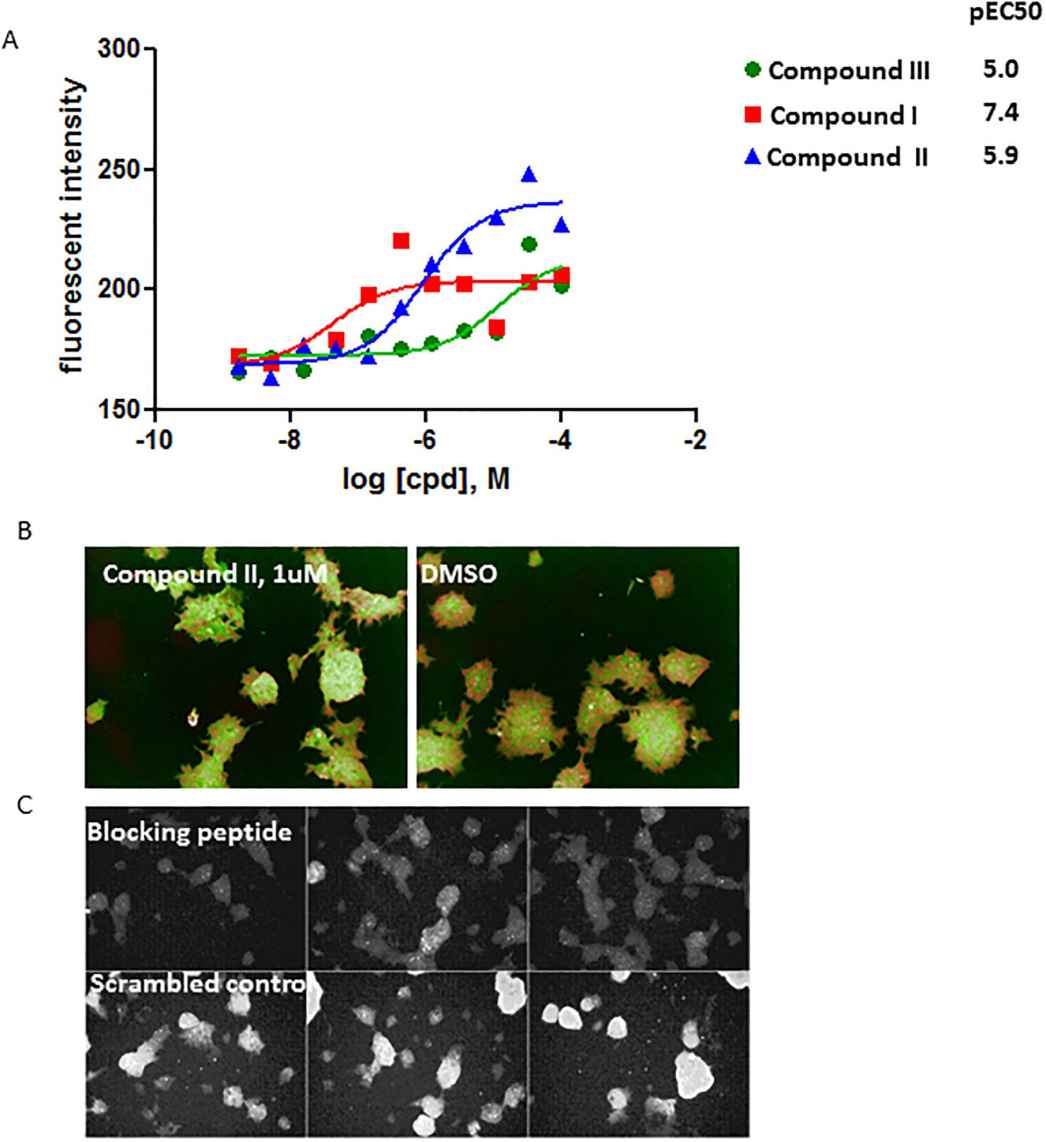
Confirmation of the hits from the Atoh1-NanoLuc reporter screen by immunofluorescent staining. Endogenous Atoh1 protein was detected using an Atoh1 antibody followed by fluorescent secondary antibody detection and counter stain by Hoechst 33342. The signal was quantified by Columbus imaging analysis system by measuring the average total intensity of the cytoplasmic signal per cell from 3 areas from each (A). Sample images of Atoh1 immunostaining in LS-174T cells treated with compound II and DMSO control (B). The Atoh1 staining (green) and the Hoechst 33342 counter-stain (red) is overlaid. The immuno-fluorescent signal of Atoh1 induced by compound II is eliminated by pre-incubating the antibody with a blocking peptide derived from the Atoh1 protein sequence (amino acid 325–354) (C), demonstrating the specificity of the antibody staining signal.

To further confirm that the *Atoh1* mRNA induced by GSIs identified from the screen correlated with Atoh1 protein expression change, we measured Atoh1 immunofluorescence staining using an Atoh1 specific antibody and performed high content imaging analysis. As shown in Fig 4A, 4B and 4C, compounds that were active in the RT-qPCR assay, namely compounds I-III, also increased Atoh1 staining in a dose dependent manner (Fig 4A and 4B and Table 1). To confirm the specificity of the signal from the Atoh1 antibody staining, the Atoh1 antibody was pre-incubated with 3 peptides derived from the immunogenic region on Atoh1 before incubation with cells for immunostaining. One peptide EENSKTSPRSHRSDGEFSPHSHYSDSDEAS spanning aa325-354 on Atoh1 protein blocked the Atoh1 immunofluorescent signal, whereas the scrambled peptide control and two other peptides covering slightly proximal regions did not (S5 Fig). These data indicate that the GSI-Notch regulated gene correlates protein level of Atoh1 in LS-174T cells. Therefore LS-174T cell is a valid model to study Notch-Atoh1 regulation.

## Discussion

Most small molecule inhibitors of Notch described today are GSIs, optimized for blocking APP processing in AD rather than for direct Notch inhibition. [3] However, Notch signaling itself remains an attractive pathway target considering its role in postnatal tissue regeneration and its association with leukemia and other non-cancer related diseases. [3] In some cases, the disease-causing mutation bypasses the requirement for Notch cleavage, such as in leukemia. [3] It is therefore important to identify modulators of Notch signaling directly and in physiologically relevant settings. We have taken advantage of the intact Notch signaling in the colon cancer cell line LS-174T and designed cell-based transcription assays to measure Notch target *Atoh1* gene expression. We demonstrated that our screening strategy, using the *Atoh1* gene promoter-driven reporter assay, in combination with the follow-up medium throughput RT-qPCR assay for endogenous *Atoh1* expression, is suitable to identify Notch inhibitors in a library consisting of thousands of compounds, thus providing a path forward for future high-throughput screening with much larger libraries, potentially containing millions of compounds. In addition to cleavage, Notch signaling has been reported to be regulated by ubiquitination, phosphorylation and hydroxylation, which provide potential additional points of intervention. [3] In addition to Notch-Hes1, which functions through the 5’ transcription regulatory region on *Atoh1* gene, it has been reported that another transcription factor, CDX2, binds to a 3’ region in *Atoh1* open reading frame and drives *Atoh1* expression in a Hes1 independent manner. [29] While the current study focused on the Notch-Hes1 regulation of Atoh1 expression, inclusion of the 3’ region in the Nano-Luc reporter in future screens may help to reveal novel mechanisms of Atoh1 regulation that target CDX2 in a Hes1 independent manner. Using the cell-based transcription assay detecting endogenous gene expression would allow confirmation of hits targeting both mechanisms in LS-174T and potentially other cell lines impinging on *Atoh1* transcription which may be useful for promoting cochlear sensory hair cell regeneration.

The source of false positives in the *Atoh1* NanoLuc reporter assay and the reason for the discrepancy between the reporter assay and the endogenous gene transcription assay are currently unknown. What is known is that luciferase reporter-based screens are prone to false positive “frequent hitters”. [34, 35] These include compounds that bind and stabilize the luciferase enzyme and inhibitors that affect cell apoptosis and differentiation. [34] Use of a mut-*Atoh1* reporter assay was effective in eliminating some but not all of these false positives. This study highlights the importance of confirming hits from reporter assays using endogenous gene transcription assays. The difference between the reporter and the endogenous promoter could be due to epigenetic regulation on the native promoter or to unidentified cis-regulatory elements embedded within the endogenous gene which do not exist on the naked synthetic promoter element. Interestingly, the reported *Atoh1* transcription regulatory element also includes a 3’ region which is regulated by Wnt signaling in addition to the 5’ region that was used in our reporter construct. This might be context dependent, as we did not observe the effect of Wnt signaling on the endogenous *Atoh1* expression in LS-174T cells.

There is a growing trend of implementing assays using human physiologically relevant tissue or cells early in drug discovery strategy which requires detection of endogenous biomarker expression. Real-time RT-qPCR assays require small sample input and can be easily adapted for any cells and any genes with minor modifications of the assay protocol. The assay can also be configured to measure multiple gene readouts to produce a more complete picture than the single gene promoter-driven reporter assay. The current bottleneck for RT-qPCR is the read time on the instrument which takes about one hour for each plate. With rapidly evolving genomics technologies, the goal of screening million compound libraries using endogenous gene transcription assays with reduced time and cost is likely achievable in the foreseeable future.

## Supporting information

S1 Fig GSI compound II, but not the Wnt signaling activating ligand R-spondin-1 induce Atoh1 expression. LS-174T cells were treated with Compound II (final concentration 1uM) or R-spondin (final concentration 2ug/ml) for 72hrs. RT-qPCR were performed as in Fig 1 and all data were normalized to DMSO control and the housekeeping gene (RPL13A) control (ΔΔCT). Results represent the mean of 2 biological duplicates and 2 PCR replicates (+/- standard deviation as error bars).
(TIF)

S2 Fig The activities of the selected compound hits identified from the ATOH1 screen. A. RT-qPCR analysis of the GSI compound treated LS-174T cells for Hes1 and ATOH1 gene expression. The expression levels are expressed as ΔCT as described in Fig 3. The compound dose on X-axis from right to left is 100, 33, 11.1, 3.7, 1.2, 0.41, 0.14, 0.05, 0.02, 0.01 and 0.00 (uM). The representative data from at least two experiments were presented. B. WT-ATOH1-NanoLuc reporter activities of representative GSI compound hits.
(TIF)

S3 Fig The effect of a subset of hit compounds from the Atoh1 reporter screen on Hes1 gene expression in RT-qPCR assay. The expression levels are expressed as ΔCT as described in Fig 3. The X-axis is the compound concentration in uM.
(TIF)

S4 Fig The activities of TACEi in the *Atoh1* RT-qPCR assay. The ADAM10 and 17 enzymatic activities were measured in fluorogenic peptide substrate assays (Reaction Biology company, Inc. Malvern PA, USA). The compounds were incubated with LS-174T cells for 72hrs as indicated doses and the endogenous gene expression of *Atoh1* were measured by RT-PCR assay as in Figs 2 and 3.
(TIF)

S5 Fig Neutralization of ATOH1 antibody by blocking peptides derived from different C-terminal regions of Atoh1. The LS-174T cells were treated with compound II at indicated doses. The Atoh1 antibody used for immunostaining was pre-incubated with or without the peptide (20x more than the antibody) for 2hrs. The immunostaining was performed as in Fig 1.
(TIF)

S1 Table Selected compound hits identified from in ATOH1 screen and the references. (TIF)

## Abbreviations

AD: Alzheimer’s disease
ADAM10 and 17: Disintegrin and metalloproteinase domain-containing protein 10 and 17
APP: Amyloid precursor Protein
Atoh1: atonal homolog 1
GSI: Gamma Secretase Inhibitor
Hes1: Hairy and enhancer of split 1
NICD: Notch intracellular domain
Notch: Neurogenic locus notch homolog protein 1
RPL13A: 60S ribosomal protein L13a
TACE: TNFαconverting enzyme
