# Supplementary figures and images for: Screen for modulators of atonal homolog 1 gene expression using notch pathway-relevant gene transcription based cellular assays
Source: PLoS One. 2018 Dec 12;13(12):e0207140. doi: 10.1371/journal.pone.0207140 (PMC6291236; doi:10.1371/journal.pone.0207140)

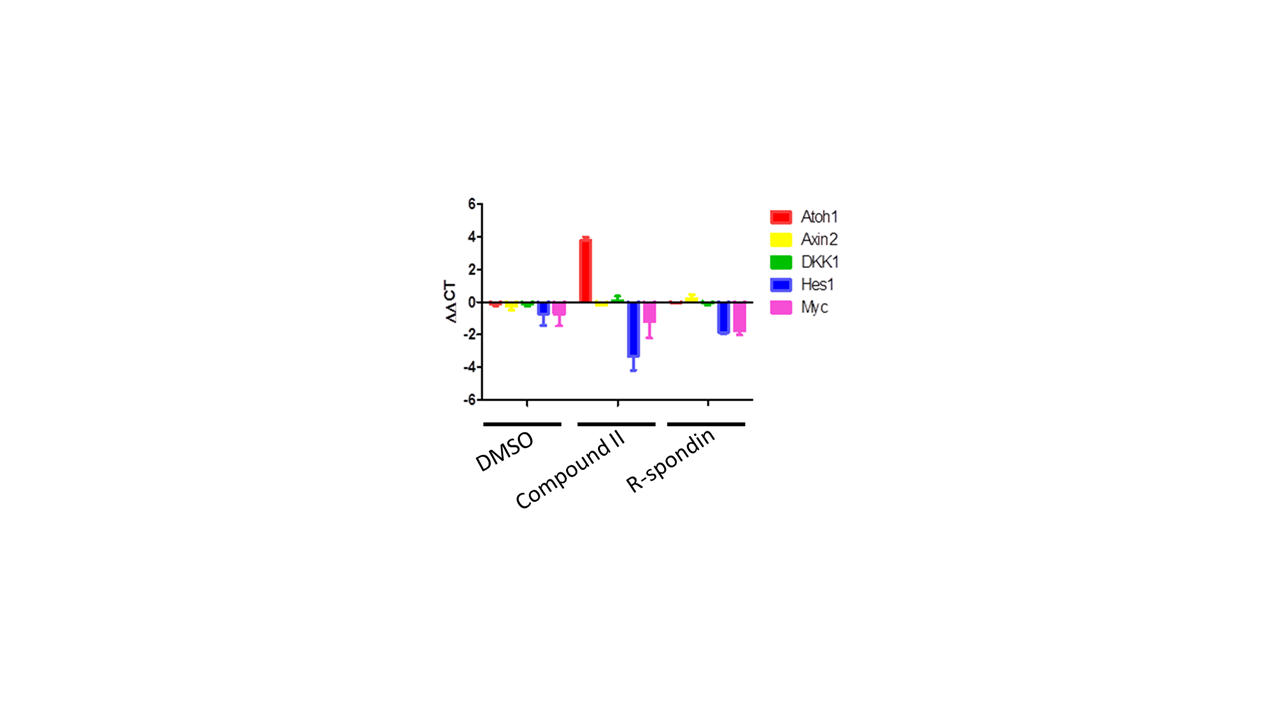

Supplement: S1 Fig — LS-174T cells were treated with Compound II (final concentration 1uM) or R-spondin (final concentration 2ug/ml) for 72hrs. RT-qPCR were performed as in Fig 1 and all data were normalized to DMSO control and the housekeeping gene (RPL13A) control (ΔΔCT). Results represent the mean of 2 biological duplicates and 2 PCR replicates (+/- standard deviation as error bars). (TIF) [file pone.0207140.s001.TIF]

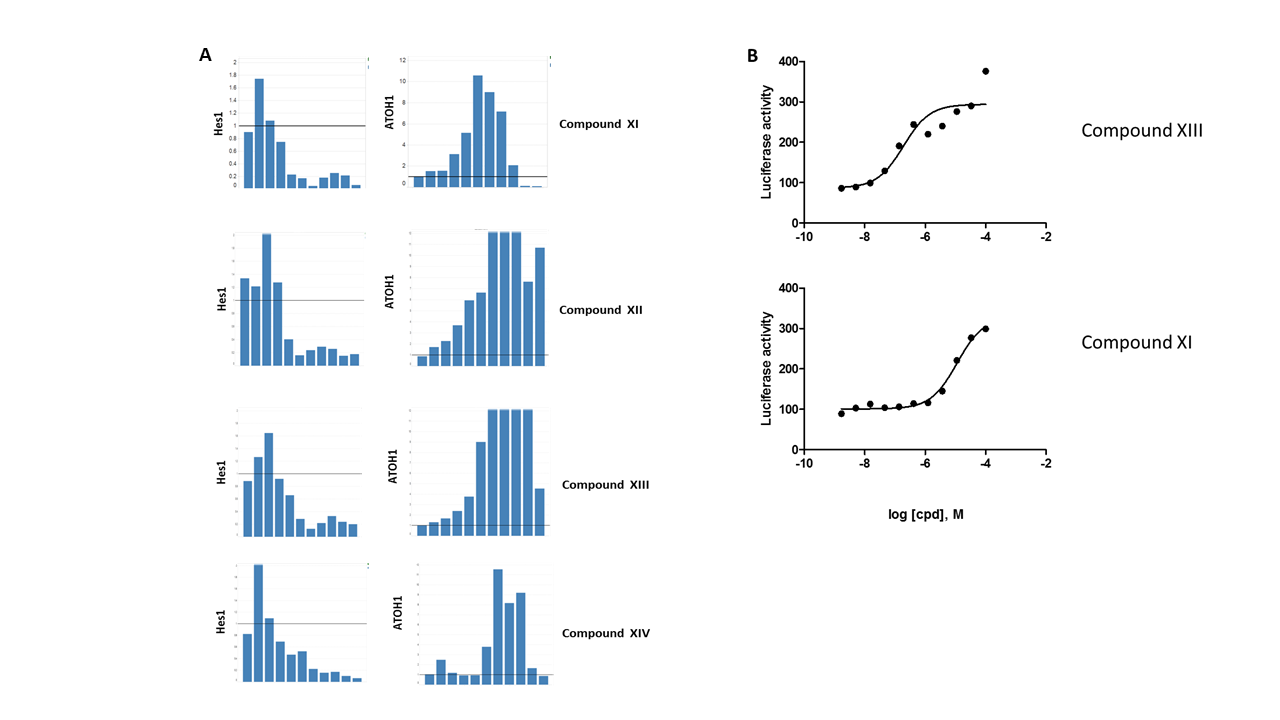

Supplement: S2 Fig — A. RT-qPCR analysis of the GSI compound treated LS-174T cells for Hes1 and ATOH1 gene expression. The expression levels are expressed as ΔCT as described in Fig 3. The compound dose on X-axis from right to left is 100, 33, 11.1, 3.7, 1.2, 0.41, 0.14, 0.05, 0.02, 0.01 and 0.00 (uM). The representative data from at least two experiments were presented. B. WT-ATOH1-NanoLuc reporter activities of representative GSI compound hits. (TIF) [file pone.0207140.s002.TIF]

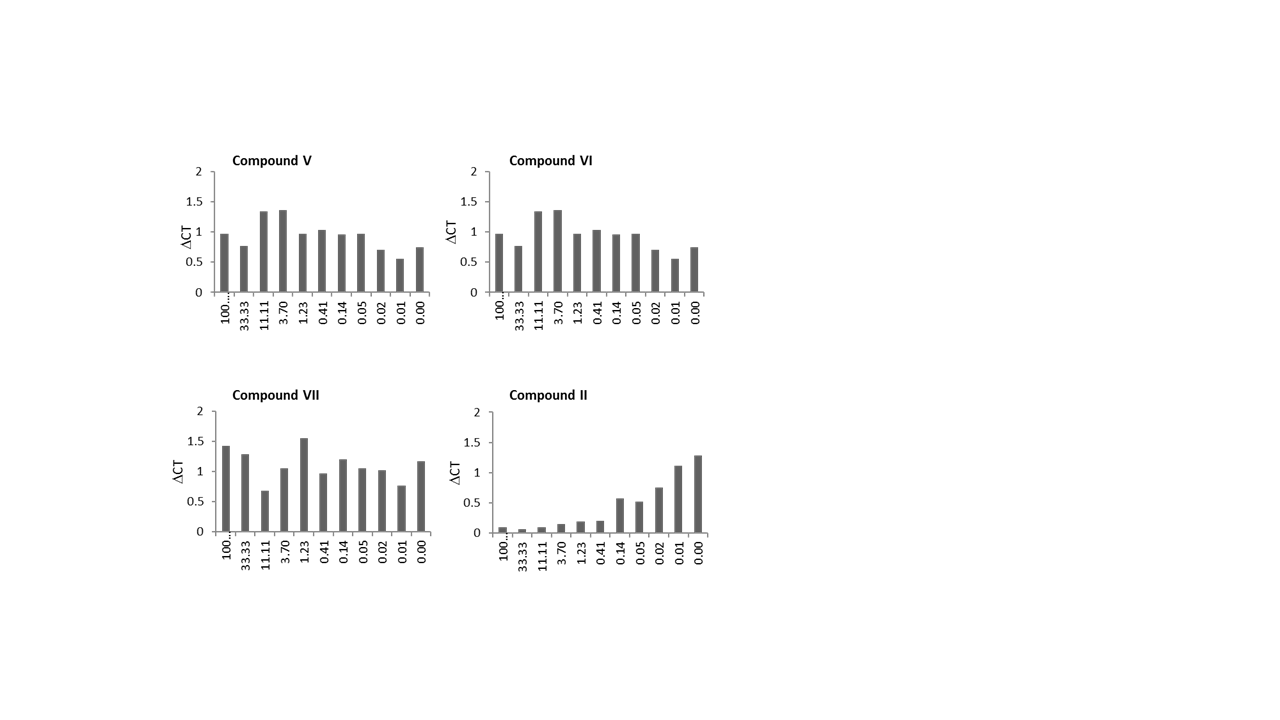

Supplement: S3 Fig — The expression levels are expressed as ΔCT as described in Fig 3. The X-axis is the compound concentration in uM. (TIF) [file pone.0207140.s003.TIF]

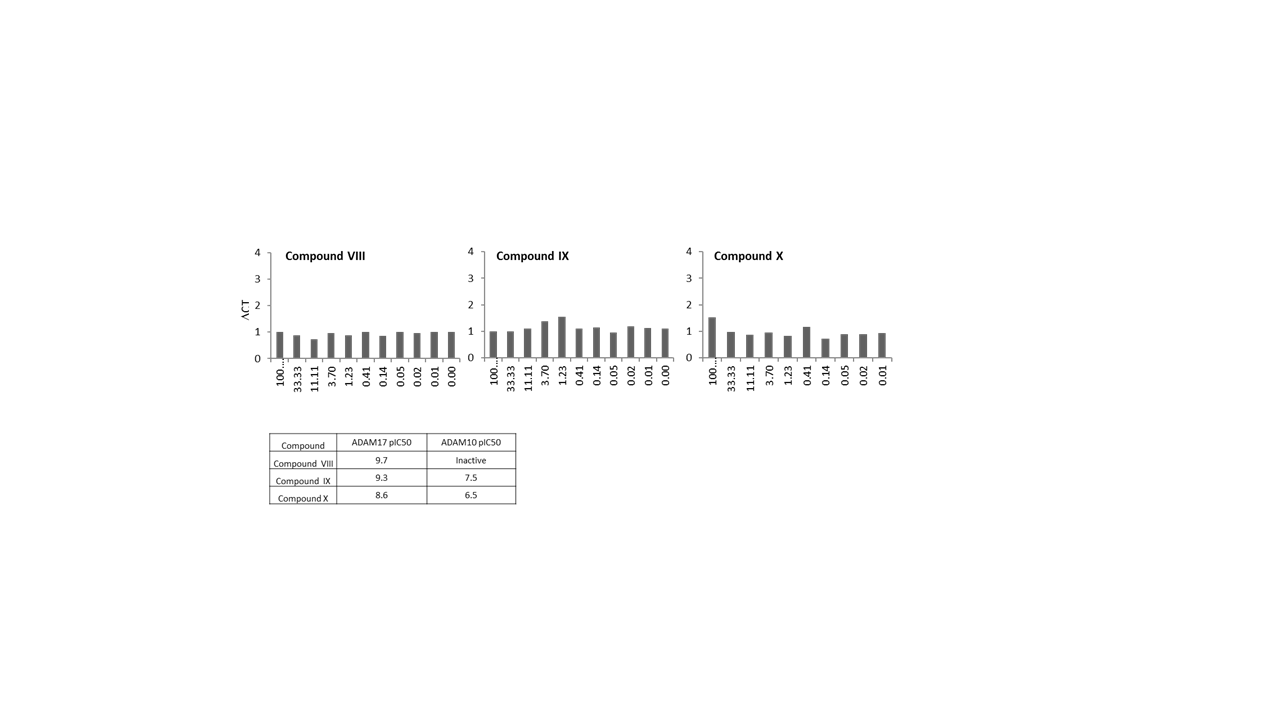

Supplement: S4 Fig — The ADAM10 and 17 enzymatic activities were measured in fluorogenic peptide substrate assays (Reaction Biology company, Inc. Malvern PA, USA). The compounds were incubated with LS-174T cells for 72hrs as indicated doses and the endogenous gene expression of Atoh1 were measured by RT-PCR assay as in Figs 2 and 3. (TIF) [file pone.0207140.s004.TIF]

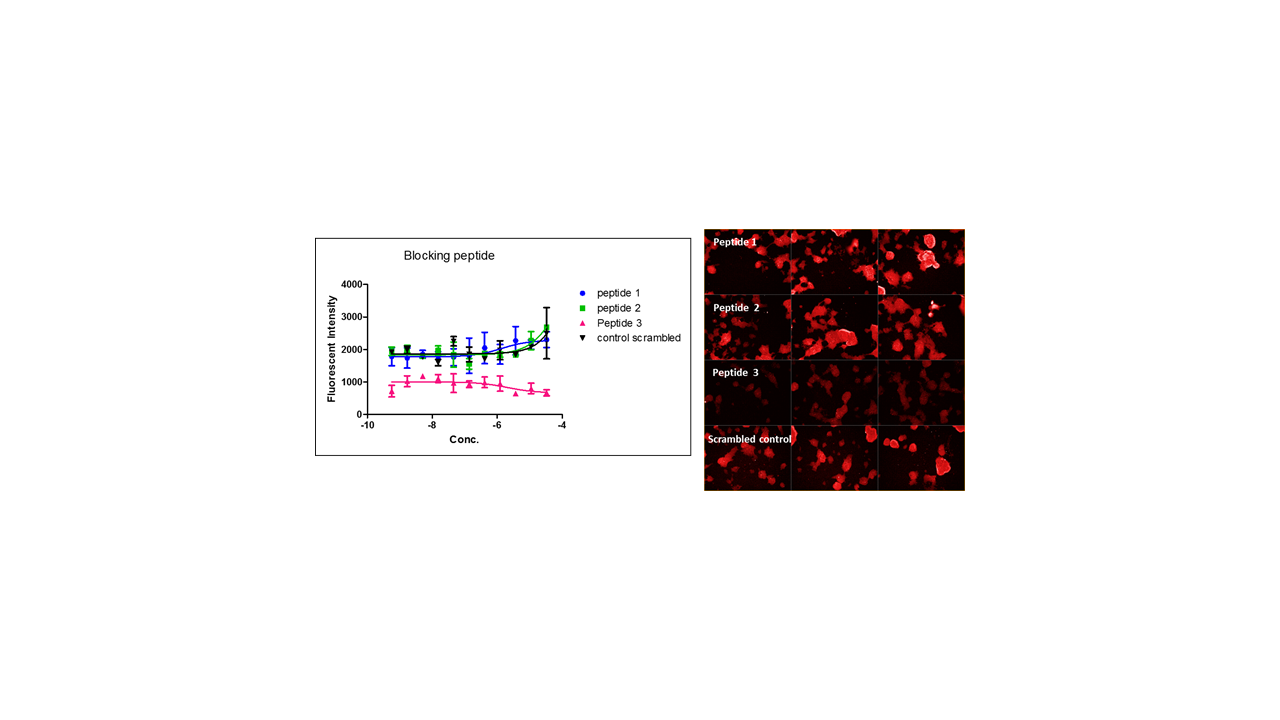

Supplement: S5 Fig — The LS-174T cells were treated with compound II at indicated doses. The Atoh1 antibody used for immunostaining was pre-incubated with or without the peptide (20x more than the antibody) for 2hrs. The immunostaining was performed as in Fig 1. (TIF) [file pone.0207140.s005.TIF]

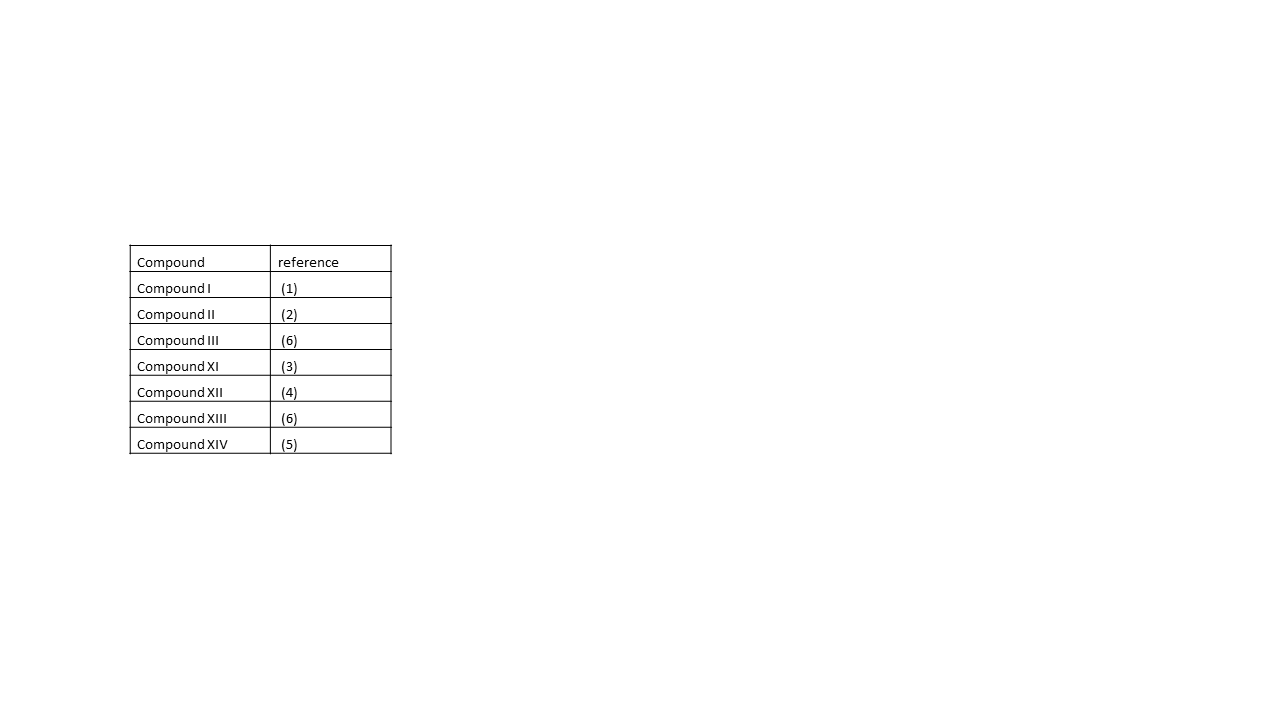

Supplement: S1 Table — (TIF) [file pone.0207140.s006.TIF]
